# Nonalcoholic Fatty Liver Disease and Risk of Early-Onset Vasomotor Symptoms in Lean and Overweight Premenopausal Women

**DOI:** 10.3390/nu14142805

**Published:** 2022-07-08

**Authors:** Yoosun Cho, Yoosoo Chang, Hye Rin Choi, Jeonggyu Kang, Ria Kwon, Ga-Young Lim, Jiin Ahn, Kye-Hyun Kim, Hoon Kim, Yun Soo Hong, Di Zhao, Sanjay Rampal, Juhee Cho, Hyun-Young Park, Eliseo Guallar, Seungho Ryu

**Affiliations:** 1 Total Healthcare Center, Kangbuk Samsung Hospital, Sungkyunkwan University School of Medicine, Seoul 04514, Korea; yoosun.cho@samsung.com; 2 Center for Cohort Studies, Total Healthcare Center, Kangbuk Samsung Hospital, Sungkyunkwan University School of Medicine, Seoul 04514, Korea; hrchoi7542@gmail.com (H.R.C.); jg1980.kang@samsung.com (J.K.); ria.kwon@samsung.com (R.K.); gayoung.lim@samsung.com (G.-Y.L.); jiin57.ahn@samsung.com (J.A.); 3 Department of Occupational and Environmental Medicine, Kangbuk Samsung Hospital, Sungkyunkwan University School of Medicine, Seoul 03181, Korea; 4 Department of Clinical Research Design & Evaluation, Samsung Advanced Institute for Health Sciences & Technology, Sungkyunkwan University, Seoul 06355, Korea; jh1448.cho@samsung.com; 5 Institute of Medical Research, Sungkyunkwan University School of Medicine, Suwon 16419, Korea; 6 Department of Obstetrics and Gynecology, Kangbuk Samsung Hospital, Sungkyunkwan University School of Medicine, Seoul 03181, Korea; khmd.kim@samsung.com; 7 Department of Obstetrics and Gynecology, Seoul National University College of Medicine, Seoul 03080, Korea; obgyhoon@gmail.com; 8 Departments of Epidemiology and Medicine, Welch Center for Prevention, Epidemiology, and Clinical Research, Johns Hopkins University Bloomberg School of Public Health, Baltimore, MD 21205, USA; hong.yunsoo@jhu.edu (Y.S.H.); dizhao@jhu.edu (D.Z.); eguallar@jhu.edu (E.G.); 9 Department of Social and Preventive Medicine, Centre for Epidemiology and Evidence Based Practice, Faculty of Medicine, Universiti Malaya, Kuala Lumpur 50603, Malaysia; srampal@ummc.edu.my; 10 Department of Precision Medicine, National Institute of Health, Korea Disease Control and Prevention Agency, Cheongju 28159, Korea; mdhypark@gmail.com

**Keywords:** menopause, vasomotor symptoms, lean, nonalcoholic fatty liver disease

## Abstract

The role of nonalcoholic fatty liver disease (NAFLD) in vasomotor symptom (VMS) risk in premenopausal women is unknown. We examined the prevalence of early-onset VMSs according to NAFLD status in lean and overweight premenopausal women. This cross-sectional study included 4242 premenopausal Korean women (mean age 45.4 years). VMSs (hot flashes and night sweats) were assessed using the Korean version of the Menopause-Specific Quality of Life questionnaire. Hepatic steatosis was determined using liver ultrasound; lean was defined as a body mass index of <23 kg/m^2^. Participants were categorized into four groups: NAFLD-free lean (reference), NAFLD-free overweight, lean NAFLD, and overweight NAFLD. Compared with the reference, the multivariable-adjusted prevalence ratios (PRs) (95% confidence intervals (CIs)) for VMSs in NAFLD-free overweight, lean NAFLD, and overweight NAFLD were 1.22 (1.06–1.41), 1.38 (1.06–1.79), and 1.49 (1.28–1.73), respectively. For moderate-to-severe VMSs, the multivariable-adjusted PRs (95% CIs) comparing NAFLD-free overweight, lean NAFLD, and overweight NAFLD to the reference were 1.38 (1.10–1.74), 1.73 (1.16–2.57), and 1.74 (1.37–2.21), respectively. NAFLD, even lean NAFLD, was significantly associated with an increased risk of prevalent early-onset VMSs and their severe forms among premenopausal women. Further studies are needed to determine the longitudinal association between NAFLD and VMS risk.

## 1. Introduction

Vasomotor symptoms (VMSs), including hot flashes and night sweats, affect approximately 80% of women experiencing menopausal transition. VMSs are bothersome symptoms and are associated with metabolic abnormalities, cardiovascular risk, and a poor quality of life [1,2,3,4,5]. They have been known to start in late perimenopause or early menopause, lasting from 6 months to 2 years [6]. However, recent studies suggest that VMSs can occur far earlier than previously reported, even during the premenopausal or early menopausal transition stages, and that they can persist for longer than 10 years after the final menstrual period [7,8]. Since early-onset and long-lasting VMSs are more likely to be related to psychological symptoms [9] and other adverse health outcomes, such as subclinical and cardiovascular events [10,11], there has been growing attention on identifying the risk factors for early-onset VMSs and their distinct pathophysiology.

A growing body of evidence suggests an association between VMSs and cardio-metabolic risk factors, including dyslipidemia, glycemic status, blood pressure, and general and central obesity, supporting VMSs as being a climacteric phenotype of metabolic syndrome [1,12]. Considering nonalcoholic fatty liver disease (NAFLD) as a hepatic manifestation of metabolic syndrome and insulin resistance, and the interrelationship between metabolic syndrome, NAFLD, and VMSs, we hypothesized that NAFLD is a risk factor for VMSs. Obesity is frequently accompanied by metabolic diseases, including NAFLD, although approximately 40% of patients with NAFLD are classified as non-obese and may have a worse prognosis than their obese counterparts [13]. Despite the distinctive features of lean NAFLD and the potential role of the independent effect of NAFLD on adverse health outcomes in the absence of obesity, no study has addressed the role of lean NAFLD and VMS risk.

In this cross-sectional study of premenopausal Korean women, we investigated the association between lean and overweight NAFLD with the prevalence of early-onset VMSs.

## 2. Materials and Methods

### 2.1. Study Population

This study used baseline data from a longitudinal study of middle-aged Korean women designed to evaluate the changes in physical and psychological health status across menopausal stages among middle-aged Korean women. Thus, Korean women aged 42–52 years in the premenopausal stage and early transition of menopausal stage were recruited between 2014 and 2018 from the Kangbuk Samsung Health Study, a cohort study of Korean adults who underwent annual or biennial comprehensive health examinations at the Kangbuk Samsung Hospital Total Healthcare Centers in Seoul and Suwon, South Korea [14,15,16]. The eligibility criteria for enrolment included the following: (1) no hormone replacement therapy or history of hysterectomy or oophorectomy; (2) at least one menstrual period in the 3 months prior to the health screening examinations and no amenorrhea lasting for ≥60 days; and (3) no history of an underlying chronic disease (malignancy, renal failure, and hypo-/hyper-thyroidism) that may affect menstrual cycles.

**Figure 1:**
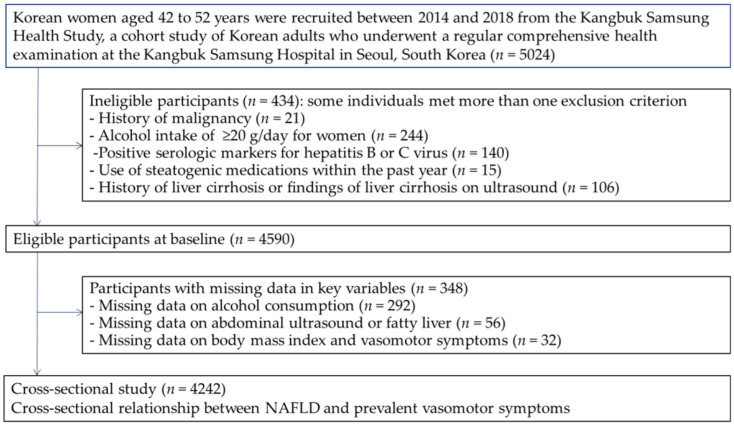
Flowchart of the study population.

Among the 5024 premenopausal women initially enrolled, we excluded participants who met one or more of the following criteria (Figure 1): (1) missing information on VMSs, body mass index, alcohol consumption, and hepatic steatosis; (2) a history of malignancy; (3) an alcohol intake of ≥20 g/day; (4) positive serologic markers for hepatitis B or C virus; (5) the use of steatogenic medications within the past year; and (6) a history of liver cirrhosis or findings of liver cirrhosis on ultrasound. The final study sample included 4242 premenopausal women.

The Institutional Review Board of Kangbuk Samsung Hospital (IRB No. KBSMC 2022-04-054) approved this study and waived the requirement for informed consent due to the use of anonymized retrospective data that were regularly collected during health screening examinations. All procedures used in this study adhered to the ethical principles of the Helsinki Declaration for Medical Research Involving Human Subjects outlined in 2013.

### 2.2. Measurements

Using standardized and self-administered questionnaires, we obtained data on demographic and reproductive characteristics and health-related behaviors. For health-related behaviors, smoking status was classified as never, former, or current smoker. Alcohol intake was categorized as an alcohol consumption of <10 or ≥10 g/day. The validated Korean version of the International Physical Activity Questionnaire short form was used to record physical activity levels, which were converted to metabolic equivalents (min/week) and classified as inactive, minimally active, or health-enhancing physical activity (HEPA) [17], defined as either ≥3 days of vigorous activities achieving at least 1500 MET min/week or ≥7 days of any combination of walking and moderate or vigorous activity achieving at least 3000 MET min/week.

Parity was categorized as nulliparous and parous, indicative of the number of pregnancies and inclusive of both live births and stillbirths. Education level was categorized as less than college graduate and equal to or greater than college graduate. Depression was defined as a self-reported physician diagnosis of depression or depression-specific medication use.

After a 5 min rest, blood pressure (BP) was measured three times using an automatic BP device (5300P, Welch Allyn, Skaneateles Falls, New York, NY, USA), and the mean BP of the second and third BP measurements was used for the analysis. Hypertension was defined as the current use of antihypertensive drugs or a BP of ≥140/90 mmHg. Blood samples were collected from the antecubital vein after at least 10 h of fasting. Fasting glucose, total cholesterol, triglyceride, high-density lipoprotein cholesterol, and low-density lipoprotein cholesterol levels were measured. Anthropometric measurements were obtained by experienced nurses, with participants wearing a lightweight hospital gown and being barefoot. Each participant’s waist circumference (WC) was measured to the nearest 0.1 cm at the halfway point between the lower margin of the rib cage and the top of the iliac crest while standing with their weight equally distributed on both feet, arms at their sides, and head straight forward. The cutoff point for abdominal obesity was defined as a WC of ≥85 cm for women according to the proposed cutoff for the Korean population [18,19].

### 2.3. Liver Ultrasound Measures and Definition of Nonalcoholic Fatty Liver Disease

As described in the exclusion criteria, we included women without secondary causes of fatty liver after excluding excessive alcohol consumption (>20 g/day) and other identifiable causes of fatty liver. Thus, fatty liver detected on ultrasonography was diagnosed as NAFLD [20]. The diagnostic criteria for fatty liver include a diffuse increase in fine echoes, deep fascicle attenuation, and bright vessel walls in the liver parenchyma compared with those in the kidney or spleen parenchyma [21]. The inter- and intra-observer reliability values for HS diagnosis were substantial (kappa statistic of 0.74) and excellent (kappa statistic of 0.94), respectively [22].

### 2.4. Definition of “Lean” or “Overweight” and Lean NAFLD vs. Overweight NAFLD

Body mass index (BMI) was calculated as body weight divided by height squared (kg/m^2^). The general international standards for defining overweight (BMI > 25 kg/m^2^) and obesity (BMI > 30 kg/m^2^) are inappropriate for Asian populations. Therefore, we adopted the BMI cutoff proposed for Asians [23]; lean was defined as a BMI of <23 kg/m^2^, and overweight was defined as a BMI of ≥23 kg/m^2^. Accordingly, the participants were categorized into four groups: NAFLD-free lean (reference), lean NAFLD, NAFLD-free overweight, and overweight NAFLD.

### 2.5. Definition of Early-Onset VMSs

VMSs include hot flashes and night sweats. Early-onset VMSs are defined as the onset of VMSs prior to menopause [24]. To assess the presence and severity of VMSs, a validated Korean version of the Menopause-Specific Quality of Life questionnaire was used [25,26]. Participants indicated whether they had experienced VMSs during the previous month and how bothersome the symptoms were on a seven-point Likert scale, ranging from “not bothered at all” (0) to “extremely bothersome” (6) [25,27]. For statistical analyses, the raw VMS intensity scores were recoded using an eight-point grading system including zero: “No” was recoded as 0 points. The severity of VMSs was recoded from 2 to 7 on a scale of 1–6. If a participant answered “No” to hot flashes or night sweats, they were regarded as having no VMSs. Participants who answered “Yes” to either hot flashes or night sweats were considered as having VMSs. Furthermore, women with one or two recoded points were classified as having mild VMSs, whereas those with ≥3 recoded points were classified as having moderate-to-severe VMSs.

### 2.6. Statistical Analysis

The characteristics of the study population were presented in four composite categories of NAFLD and BMI status: NAFLD-free lean (reference), lean NAFLD, NAFLD-free overweight, and overweight NAFLD. All data are presented as means (standard deviations), medians (interquartile ranges), or numbers (percentages), as appropriate.

Logistic regression models (with the margin command in Stata) were used to estimate prevalence ratios (PRs) and 95% confidence intervals (CIs) for prevalent VMSs (overall and their severe form, separately), comparing lean NAFLD, NAFLD-free overweight, and overweight NAFLD, with NAFLD-free lean as the reference group. We adjusted the model for potential confounders that might affect the relationship between NAFLD and VMSs. The confounding variables were defined as (1) being associated with the exposure (lean and overweight NAFLD), (2) being associated with the outcome (VMS), and (3) not belonging to the causal pathway between the exposure (lean and overweight NAFLD) and outcome (VMS). The models were initially adjusted for age and then further adjusted for BMI (continuous), education level (less than college graduate, equal to or higher college graduate, or unknown), parity (yes, no, or unknown), age at menarche (continuous), physical activity (inactive, minimally active, health-enhancing physical activity, or unknown), smoking status (never, former, current smoker, or unknown), alcohol consumption (<10 or ≥10 g/day), history of hypertension (yes, no, or unknown), history of diabetes (yes, no, or unknown), and medication use for dyslipidemia (yes, no, or unknown).

Furthermore, we performed an additional analysis for the prevalence of each VMS component (hot flashes and night sweats, separately). In a sensitivity analysis, we used abdominal obesity based on WC instead of overall obesity based on BMI, and we compared the prevalence of VMSs according to the combination of abdominal obesity and NAFLD. We performed an additional analysis to assess the association between NAFLD and the prevalence of VMS according to the fat mass percentage measured using a multi-frequency bioimpedance analyzer with eight-point tactile electrodes (InBody 720, Biospace Co., Seoul, Korea). This technique has been validated for body composition assessment and correlates well with results obtained using dual-energy X-ray absorptiometry or abdominal computed tomography [28,29]. We defined obesity using a body fat percentage cut-off of ≥35% for women [30].

We also defined NAFLD severity using the Fibrosis-4 index (FIB-4), a validated non-invasive index of advanced fibrosis, to evaluate the severity of hepatic steatosis [31]. The FIB-4 cut-off points were defined as <1.30 (low risk), 1.30–2.67 (intermediate risk), and ≥2.67 (high risk) to predict the probability of advanced fibrosis [31].

Additionally, we conducted a prospective cohort study to assess the risk of incident VMS according to NAFLD status in lean and overweight women without VMS at baseline who had at least one follow-up VMS assessment before December 2021 (*n* = 2349) (Figure S1). Cox proportional hazards models were used to calculate adjusted hazard ratios (aHRs) and 95% confidence intervals (CIs) for incident VMS according to NAFLD status in lean and overweight women after adjusting for confounders. The risk of incident VMS according to NAFLD status was also estimated based on different adiposity measures, such as WC and body fat percentage.

All statistical analyses were performed using Stata version 17.0 (Stata Corp LP; College Station, TX, USA). Statistical significance was defined as a two-sided *p*-value of <0.05.

## 3. Results

The mean age of the 4242 women included in this study was 45.4 (SD 2.7) years (Table 1). The overall prevalence of VMSs and NAFLD was 22.0% and 18.9%, respectively. The proportion of women in the premenopausal stage and early menopausal transition was 94.6% and 5.4%, respectively. Women with NAFLD were more likely to have VMSs and metabolic diseases, including hypertension, diabetes, and dyslipidemia, than those without NAFLD, among both lean and overweight individuals, and they were less likely to be physically active. The prevalence of depression in our study population was 1.91%. Women with NAFLD seemed less likely to have depression than those without NAFLD. Regardless of how obesity was defined (WC or BMI), the patterns in baseline characteristics did not differ (Table S1).

Table 2 shows the cross-sectional association of the prevalence of overall VMSs and their severe form (moderate-to-severe VMSs) according to the composite categories of NAFLD and BMI status. The prevalence of overall VMSs was 18.89% for NAFLD-free lean, 26.79% for lean NAFLD, 23.78% for NAFLD-free overweight, and 30.6% for overweight NAFLD. After adjustment for potential confounders, including age; education level; parity; physical activity level; smoking status; alcohol intake; age of menarche; and history of hypertension, diabetes, and dyslipidemia, the multivariable-adjusted PRs (95% CIs) for prevalent VMSs comparing NAFLD-free overweight, lean NAFLD, and overweight NAFLD to NAFLD-free lean (reference) were 1.22 (1.06–1.41), 1.38 (1.06–1.79), and 1.49 (1.28–1.73), respectively. For moderate-to-severe VMS prevalence, the multivariable-adjusted PRs (95% CIs) in NAFLD-free overweight, lean NAFLD, and overweight NAFLD were 1.38 (1.10–1.74), 1.73 (1.16–2.57), and 1.74 (1.37–2.21), respectively, compared with the reference (Table 2). However, in the post hoc comparison test, there was no significant difference in the prevalence of VMSs between lean NAFLD and NAFLD-free overweight. When we added depression as another covariate and included it in the multivariate analysis, the results remained consistent with our main findings (Table S2).

## 4. Discussion

In the analyses using abdominal obesity based on WC instead of BMI, abdominal non-obese NAFLD was significantly associated with overall VMSs (PR 1.23, 95% CI 1.03–1.47), moderate-to-severe VMSs (PR 1.59, 95% CI 1.21–2.08), and each VMS component (PR 1.26, 95% CI 1.03–1.53 and PR 1.41, 95% CI 1.11–1.78 for hot flashes and night sweats, respectively) (Tables S3 and S4). NAFLD-free abdominal obesity was significantly associated with moderate-to-severe VMSs (PR 1.73, 95% CI 1.19–2.51) but not overall VMSs (PR 1.16, 95% CI 0.90–1.51). Using fat mass percentage instead of BMI or WC as another proxy for obesity, NAFLD was consistently associated with an increased prevalence of overall and moderate-to-severe VMSs, even in women with a fat mass percentage of <35% (non-obese range) (PR 1.24, 95% CI 1.01–1.51 and PR 1.56, 95% CI 1.15–2.21, respectively) (Table S5).

Then, we further evaluated the association between NAFLD severity based on FIB-4 and VMSs (Table S6). However, in our study comprising relatively healthy middle-aged women, most patients with NAFLD (97%) had low FIB-4 scores, and only two patients with lean NAFLD had an intermediate/high FIB-4 score. NAFLD with an intermediate/high FIB-4 score tended to be associated with the highest prevalence of VMSs, but this association was only significant in the overweight NAFLD group (PR 2.33, 95% CI 1.40–3.89).

During a median follow-up of 4.2 years (interquartile range 3.2–5.2 years), 191 women with NAFLD developed VMSs. Compared with NAFLD-free lean, overweight NAFLD significantly increased the risk of incident VMSs (HR 1.31, 95% CI 1.09–1.59), whereas lean NAFLD tended to have an increased risk of VMSs, although this association did not reach statistical significance (HR 1.19, 95% CI 0.85–1.65) (Table S7). An investigation of the association between NAFLD according to other adiposity measures (WC and body fat percent) and incident VMSs revealed no significant associations.

In the present cross-sectional study of premenopausal women, both lean and overweight NAFLD were consistently associated with an increased prevalence of overall VMSs (hot flashes and night sweats) and moderate-to-severe VMSs compared with NAFLD-free lean. The highest prevalence of VMSs was observed in overweight NAFLD. Furthermore, WC-defined abdominal non-obese NAFLD was consistently associated with an increased risk of VMSs, including overall VMSs, moderate-to-severe VMSs, and each component. Our findings suggest that NAFLD is an independent risk factor for VMSs, even in the absence of general and abdominal obesity.

However, studies on the association between NAFLD and VMSs are limited. A cross-sectional study of 1793 postmenopausal Korean women aged 45–64 years demonstrated a significant positive association between NAFLD and moderate-to-severe VMSs after adjusting for confounders, including central obesity and insulin resistance [32]. Overweight/obesity is an important risk factor for VMSs and NAFLD. Although the effect of BMI on VMSs differs among the pre-, peri-, and post-menopausal stages [33], overweight/obesity is generally known to be associated with an increased risk of VMSs [34,35,36]. NAFLD is commonly accompanied by obesity, and it is important to determine whether NAFLD, irrespective of excessive adiposity, plays an independent role in the development of VMSs. Indeed, the increased risk of VMSs in obese women can be explained by the insulation effect of the increased fat layer against heat dissipation, leading to an increased core body temperature [37]. In our study of exclusively premenopausal women, an independent association between NAFLD and an increased prevalence of VMSs was consistently observed, even in lean individuals; thus, the observed association could not be explained by general obesity. Central obesity, which increases with visceral adiposity, appears to be closely associated with insulin resistance, which modulates VMS risk in NAFLD by releasing proinflammatory cytokines [38]. However, in our study, NAFLD was associated with an increased risk of VMSs, regardless of general or abdominal obesity; both lean NAFLD and abdominal non-obese NAFLD were associated with an increased risk of overall VMSs and their severe forms. Our findings support VMSs as a climacteric phenotype of unhealthy metabolic status, indicated by the coexistence of NAFLD that is not simply confounded by overall and abdominal obesity.

For specific VMS components (hot flashes and night sweats), the NAFLD-free overweight category was only associated with hot flashes but not night sweats, whereas lean NAFLD and overweight NAFLD categories were significantly associated with both symptoms. Night sweats could be a manifestation of the most severe hot flashes or a symptom that is more difficult to endure than hot flashes [39]. Some previous studies support that both hot flashes and night sweats may increase the risk of adverse health outcomes, such as cardiovascular disease [24,40]; another study found a greater increase in cardiovascular disease risk associated with night sweats compared with hot flashes [39,41]; and an increased risk of incident diabetes was profoundly observed in women reporting night sweats regardless of accompanying hot flashes [42]. Accordingly, the risk of VMSs with NAFLD may have a relatively greater clinical implication than VMS risk in overweight women, especially for cardio-metabolic health. Further studies are required to examine the distinct mechanisms underlying the association between obesity, NAFLD, and different VMS subtypes.

Several possible mechanisms have been suggested for the relationship between VMSs and NAFLD. Due to unpredictable variability or acute reduction in estrogen levels, an alteration in the thermoregulatory system through a sympathetic pathway leads to rapid and frequent heat-dissipating reactions, which is a key pathogenesis of VMSs [43]. Metabolic abnormalities, such as NAFLD, are also related to hyperactivity of the hypothalamic–pituitary–adrenal axis, which may partially explain the association between NAFLD and VMS risk [44,45]. The overexpression of catecholamines, norepinephrine, epinephrine, or neuropeptide Y under the activation of intracellular signaling pathways in hepatic stellate cells has been observed in human NAFLD cells and is involved in hepatic fibrogenesis and inflammation [46]. Moreover, increased cortisol levels, a reliable index of hypothalamic–pituitary–adrenal axis activity, are associated with VMSs [47] and may also be implicated in NAFLD development [48]. Further longitudinal studies are required to determine the temporal relationship between NAFLD and VMSs and the underlying mechanisms.

Our study has several limitations. First, our study was cross-sectional, and no causal relationship between NAFLD and VMSs was established. Second, VMSs were assessed using a self-administered questionnaire, which could lead to misclassification due to recall bias. Third, we did not perform a liver biopsy, which is the gold standard for NAFLD diagnosis. Since it is neither ethical nor feasible to obtain histological data on HS and fibrosis from liver biopsies of relatively healthy participants, we used a noninvasive diagnosis of fatty liver using ultrasonography, which has been validated with acceptable accuracy and reproducibility and is widely used in population-based studies [49,50]. Fourth, data on serum estrogen levels were not available in the present study. Additional information on the hormonal levels in each group, with the composition of NAFLD and BMI, suggests the pathophysiology underlying our findings. Finally, since our study population consisted of healthy middle-aged Korean women who were able to easily access health care facilities, the findings of our study may not be generalizable to other populations with different ethnicities or demographic characteristics.

## 5. Conclusions

In this large-scale study of premenopausal Korean women exclusively in the late reproductive stage or early menopausal transition stage, NAFLD was found to be significantly associated with an increased risk of prevalent overall VMSs, each component (hot flashes and night sweats), and their severe forms, regardless of general and abdominal obesity. Our findings suggest that even lean individuals with NAFLD are at an increased risk of VMSs, and further studies with longitudinal designs are needed to confirm whether NAFLD is an independent risk factor for VMS development.

Considering that NAFLD is a hepatic manifestation of metabolic syndrome and insulin resistance, and that VMSs are correlated with these diseases, we expect that identifying risk factors for VMSs, such as NAFLD, may lead to effective management. As an alternative to hormonal replacement therapy, lifestyle modifications with weight reduction, diet, and regular exercise have been suggested as practical nonpharmacological approaches to alleviate VMSs, including hot flashes and night sweats [51]. Recommended as one of the most evidence-based interventions to relieve VMSs [52], weight reduction is the most critical intervention for NAFLD, even for lean individuals [53]. Taken together, physicians need to remember that women with NAFLD, irrespective of obesity, could be at risk of VMSs. Further longitudinal studies are needed to determine whether the resolution of NAFLD independently reduces the risk of incident VMSs and whether weight reduction mediates or confounds the association.

## Figures and Tables

**Table 1:** Baseline characteristics of study participants (*n* = 4242).

Characteristics	Overall		Lean		Overweight	
	NAFLD (−)	NAFLD (+)	NAFLD (−)	NAFLD (+)	NAFLD (−)	NAFLD (+)
Number of participants	3440	802	2536	168	904	634
Vasomotor symptoms (%)	694 (20.17)	239 (29.80)	479 (18.89)	45 (26.79)	215 (23.78)	194 (30.60)
Age (years) *	44.79 (2.42)	45.42 (2.65)	44.66 (2.36)	45.12 (2.60)	45.17 (2.56)	45.50 (2.67)
Age of menarche	13.95 (1.39)	13.90 (1.41)	14.00 (1.36)	14.15 (1.34)	13.78 (1.45)	13.83 (1.42)
Parity (%)	3056 (92.44)	713 (92.60)	2242 (92.07)	143 (91.67)	814 (93.46)	570 (92.83)
Menopausal stage						
Premenopausal (%)	3257 (94.68)	761 (94.89)	2400 (94.64)	160 (95.24)	857 (94.80)	601 (94.79)
Transitional, early (%)	183 (5.32)	41 (5.11)	136 (5.36)	8 (4.76)	47 (5.20)	33 (5.21)
Current smoker (%)	392 (11.40)	76 (9.48)	296 (11.67)	20 (11.90)	96 (10.62)	56 (8.83)
Alcohol intake (%) ^†^	275 (7.99)	77 (9.60)	188 (7.41)	18 (10.71)	87 (9.62)	59 (9.31)
Physical activity level (%) ^‡^						
inactive	1754 (51.26)	446 (55.89)	1303 (51.62)	93 (56.02)	451 (50.22)	353 (55.85)
minimally active	1138 (33.26)	251 (31.45)	845 (33.48)	54 (32.53)	293 (32.62)	197 (31.17)
HEPA	530 (15.49)	101 (12.66)	376 (14.90)	19 (11.45)	154 (17.15)	82 (12.97)
Higher education level (%) ^||^	2770 (82.00)	612 (77.18)	2081 (83.47)	134 (80.24)	689 (77.85)	478 (76.36)
Depression (%)	68 (1.98)	13 (1.62)	52 (2.05)	3 (1.79)	16 (1.77)	10 (1.58)
Hypertension (%)	119 (3.46)	87 (10.88)	63 (2.49)	7 (4.17)	56 (6.19)	80 (12.66)
Diabetes (%)	30 (0.87)	58 (7.23)	18 (0.71)	8 (4.76)	12 (1.33)	50 (7.89)
Medication for dyslipidemia	37 (1.08)	36 (4.49)	23 (0.91)	2 (1.19)	14 (1.55)	34 (5.36)
BMI (kg/m^2^) *	21.71 (2.43)	25.52 (3.13)	20.59 (1.42)	21.73 (0.97)	24.84 (1.84)	26.52 (2.72)
Systolic BP (mmHg) *	102.34 (10.83)	109.35 (12.87)	100.86 (10.13)	103.09 (10.58)	106.48 (11.61)	111.01 (12.93)
Diastolic BP (mmHg) *	65.82 (8.73)	70.02 (9.61)	65.14 (8.52)	66.70 (8.67)	67.75 (9.04)	70.90 (9.66)
Glucose (mg/dL) ^*^	91.55 (9.46)	99.61 (19.78)	90.94 (9.35)	94.43 (10.63)	93.28 (9.55)	100.98 (21.36)
Total cholesterol (mg/dL) *	191.37 (29.64)	200.91 (31.53)	190.58 (29.59)	201.08 (30.38)	193.61 (29.70)	200.86 (31.86)
LDL-C (mg/dL) *	116.28 (27.13)	132.36 (30.07)	114.21 (26.68)	129.46 (30.05)	122.13 (27.54)	133.13 (30.05)
HDL-C (mg/dL) *	68.81 (15.17)	56.13 (13.54)	70.66 (15.30)	60.90 (14.79)	63.63 (13.53)	54.86 (12.91)
Triglycerides (mg/dL) ^§^	77.78 (34.53)	119.54 (61.15)	74.47 (31.67)	108.20 (65.16)	87.07 (40.09)	122.54 (59.74)

Data are presented as * mean (± standard deviation), ^§^ median (interquartile range), or percentage. Abbreviations: BMI, body mass index; BP, blood pressure; HDL-C, high-density lipoprotein cholesterol; LDL-C, low-density lipoprotein cholesterol; NAFLD, nonalcoholic fatty liver disease; HEPA, health-enhancing physical activity. ^†^ ≥10 g of ethanol per day ^‡^ ≥3 times/week. ^||^ ≥ college graduate.

**Table 2:** Cross-sectional association between nonalcoholic fatty liver disease and vasomotor symptoms in lean and overweight premenopausal women (*n* = 4242).

Vasomotor Symptoms		Lean		Overweight	
		NAFLD (−)	NAFLD (+)	NAFLD (−)	NAFLD (+)
Overall	No. of cases	479	45	215	194
	Prevalence (%)	18.89	26.79	23.78	30.60
	Age-adjusted PR * (95% CI)	reference	1.39 (1.08–1.81)	1.24 (1.07–1.42)	1.57 (1.36–1.82)
	Multivariable-adjusted PR *^,^^†^ (95% CI)	reference	1.38 (1.06–1.79)	1.22 (1.06–1.41)	1.49 (1.28–1.73)
Moderate- to-severe	No. of cases	198	24	101	96
	Prevalence (%)	7.81	14.29	11.18	15.14
	Age-adjusted PR * (95% CI)	reference	1.79 (1.21–2.65)	1.40 (1.12–1.76)	1.87 (1.49–2.36)
	Multivariable-adjusted PR *^,^^†^ (95% CI)	reference	1.73 (1.16–2.57)	1.38 (1.10–1.74)	1.74 (1.37–2.21)

* Logistic regression models with robust variance were used to estimate PRs and 95% CIs for prevalent vasomotor symptoms. ^†^ The multivariable model was adjusted for age; education level; parity; physical activity level; smoking status; alcohol intake; age at menarche; and history of hypertension, diabetes, and dyslipidemia. Abbreviations: CI, confidence interval; NAFLD, nonalcoholic fatty liver disease; PR, prevalence ratio. In the sensitivity analysis, where each VMS component of hot flashes and night sweats was used as a dependent variable (Table 3), lean and overweight NAFLD were consistently associated with increased prevalence of hot flashes (PR 1.42, 95% CI 1.06–1.91 vs. PR 1.60, 95% CI 1.35–1.88) and night sweats (PR 1.44, 95% CI 1.02–2.04 vs. PR 1.54, 95% CI 1.26–1.88) compared with NAFLD-free lean. NAFLD-free overweight was significantly associated with increased prevalence of hot flashes (PR 1.28, 95% CI 1.09–1.50) but not night sweats (PR 1.12, 95% CI 0.92–1.36).

**Table 3:** Cross-sectional association between nonalcoholic fatty liver disease and vasomotor symptoms (hot flashes or night sweats) in lean and overweight premenopausal women (*n* = 4242).

		Lean		Overweight	
		NAFLD (−)	NAFLD (+)	NAFLD (−)	NAFLD (+)
	No. of cases	394	38	185	170
	Prevalence (%)	15.53	22.62	20.49	26.86
Hot flashes	Age-adjusted PR * (95% CI)	reference	1.44 (1.07–1.93)	1.30 (1.11–1.52)	1.69 (1.44–1.99)
	Multivariable-adjusted PR ^*^ (95% CI)	reference	1.42 (1.06–1.91)	1.28 (1.09–1.50)	1.60 (1.35–1.88)
	No. of cases	302	30	122	125
	Prevalence (%)	11.84	17.75	13.54	19.81
Night sweats	Age-adjusted PR * (95% CI)	reference	1.48 (1.05–2.07)	1.13 (0.92–1.37)	1.63 (1.34–1.97)
	Multivariable-adjusted PR *^,†^ (95% CI)	reference	1.44 (1.02–2.04)	1.12 (0.92–1.36)	1.54 (1.26–1.88)

* Logistic regression models with robust variance were used to estimate PRs and 95% Cis for prevalent vasomotor symptoms. ^†^ The multivariable model was adjusted for age; education level; parity; physical activity level; smoking status; alcohol intake; age at menarche; and history of hypertension, diabetes, and dyslipidemia. Abbreviations: CI, confidence interval; NAFLD, nonalcoholic fatty liver disease; PR, prevalence ratio.

## Data Availability

The data presented in this study are available on request from the corresponding author. The data are not publicly available due to ethical requirements.

## Supplementary Materials

The following are available online at https://www.mdpi.com/article/10.3390/nu14142805/s1, Figure S1: Flow chart of the study population (*n* = 2349), Table S1: Cross-sectional association between nonalcoholic fatty liver disease and vasomotor symptoms in premenopausal women according to abdominal obesity (*n* = 4240), Table S2: Cross-sectional association between nonalcoholic fatty liver disease and vasomotor symptoms in lean and overweight premenopausal women after adjusting for confounders including depression (*n* = 4242), Table S3: Cross-sectional association between nonalcoholic fatty liver disease and vasomotor symptoms in premenopausal women according to abdominal obesity (*n* = 4240), Table S4: Cross-sectional association between nonalcoholic fatty liver disease and vasomotor symptoms (hot flashes or night sweats) in premenopausal women according to abdominal obesity (*n* = 4240), Table S5: Cross-sectional association between nonalcoholic fatty liver disease and vasomotor symptoms in premenopausal women according to body fat percentage (*n* = 4231), Table S6: Cross-sectional association between nonalcoholic fatty liver disease with or without intermediate-to-high probability of advanced fibrosis based on the Fibrosis-4 index and vasomotor symptoms in premenopausal women according to general obesity (*n* = 4232), Table S7: Longitudinal association between nonalcoholic fatty liver disease and vasomotor symptoms in premenopausal women according to adiposity measures.
