# Supplementary material for: Nonalcoholic Fatty Liver Disease and Risk of Early-Onset Vasomotor Symptoms in Lean and Overweight Premenopausal Women
Source: Nutrients. 2022 Jul 8;14(14):2805. doi: 10.3390/nu14142805 (PMC9317337; doi:10.3390/nu14142805)

Nonalcoholic fatty liver disease and risk of early-onset vasomotor symptoms in lean and overweight premenopausal women

Yoosun Cho[a], Yoosoo Chang [b,c,d*], Hye Rin Choi[b], Jeonggyu Kang[b], Ria Kwon[b], Gayoung Lim[b], Jiin Ahn[b], Kye-Hyun Kim[e], Hoon Kim[f], Yunsoo Hong[g], Di Zhao[g], Sanjay Rampal[h], Juhee Cho[a,c,g], Hyun-Young Park[i], Eliseo Guallar[g], Seungho Ryu[a,b,c,*]

Supplementary Tables

Table S1. Cross-sectional association between nonalcoholic fatty liver disease and vasomotor symptoms in premenopausal women according to abdominal obesity (n = 4,240)

Table S2. Cross-sectional association between nonalcoholic fatty liver disease and vasomotor symptoms in lean and overweight premenopausal women after adjusting for confounders including **depression** (n = 4,242)

Table S3. Cross-sectional association between nonalcoholic fatty liver disease and vasomotor symptoms in premenopausal women according to abdominal obesity (n = 4,240)

Table S4. Cross-sectional association between nonalcoholic fatty liver disease and vasomotor symptoms (hot flashes or night sweats) in premenopausal women according to abdominal obesity (n = 4,240)

Table S5. Cross-sectional association between nonalcoholic fatty liver disease and vasomotor symptoms in premenopausal women according to body fat percentage (n = 4,231)

Table S6. Cross-sectional association between nonalcoholic fatty liver disease with or without intermediate-to-high probability of advanced fibrosis based on the Fibrosis-4 index and vasomotor symptoms in premenopausal women according to general obesity (n = 4,232)

Table S7. Longitudinal association between nonalcoholic fatty liver disease and vasomotor symptoms in premenopausal women according to adiposity measures

Figure S1. Flow chart of the study population (n = 2,349)

Table S1. Baseline characteristics of the study participants according to abdominal obesity (n = 4,240)

Characteristics	Overall		Abdominal non-obese		Abdominal obese	
	NAFLD (−)	NAFLD (+)	NAFLD (−)	NAFLD (+)	NAFLD (−)	NAFLD (+)
Number of participants	3,438	802	3,234	484	204	318
Vasomotor symptoms (%)	694 (20.19)	239 (29.80)	640 (19.79)	131 (27.07)	54 (26.47)	108 (33.96)
Age (years) *	44.79 (2.42)	45.42 (2.65)	44.76 (2.40)	45.47 (2.67)	45.31 (2.69)	45.34 (2.63)
Age of menarche	13.95 (1.39)	13.90 (1.41)	13.96 (1.37)	14.01 (1.40)	13.78 (1.58)	13.74 (1.41)
Parity (%)	3,055 (92.44)	713 (92.60)	2,874 (92.47)	436 (93.36)	181 (91.88)	277 (91.42)
Menopausal stage						
Premenopausal (%)	3,256 (94.71)	761 (94.89)	3,060 (94.64)	453 (93.60)	196 (96.08)	308 (96.86)
Transitional, early (%)	182 (5.29)	41 (5.11)	174 (5.38)	31 (6.40)	8 (3.92)	10 (3.14)
Current smoker (%)	392 (11.40)	76 (9.48)	365 (11.29)	44 (9.09)	27 (13.24)	32 (10.06)
Alcohol intake (%)†	275 (8.00)	77 (9.60)	252 (7.79)	48 (9,92)	23 (11.27)	29 (9.12)
Physical activity level						

(%)‡						
Inactive	1,753 (51.26)	446 (55.89)	1,644 (51.09)	260 (54.17)	109 (53.96)	186 (58.49)
Minimally active	1,138 (33.27)	251 (31.45)	1,084 (33.69)	157 (32.71)	54 (26.73)	94 (29.56)
HEPA	529 (15.47)	101 (12.66)	490 (15.23)	63 (13.13)	39 (19.31)	38 (11.95)
Higher education level (%)‖	2,769 (82.00)	612 (77.18)	2,620 (82.39)	376 (78.66)	149 (75.63)	236 (74.92)
Depression (%)	68 (1.98)	13 (1.62)	65 (2.01)	6 (1.24)	3 (1.47)	7 (2.20)
Hypertension (%)	119 (3.46)	87 (10.88)	109 (3.37)	41 (8.49)	10 (4.90)	46 (14.51)
Diabetes (%)	30 (0.87)	58 (7.23)	24 (0.74)	23 (4.75)	6 (2.94)	35 (11.01)
Medication for dyslipidemia	37 (1.08)	36 (4.49)	34 (1.05)	16 (3.31)	3 (1.47)	20 (6.29)
BMI (kg/m^2) *	21.71 (2.43)	25.52 (3.13)	21.39 (2.05)	23.81 (1.94)	26.70 (2.55)	28.12 (2.80)
Systolic BP (mmHg) *	102.34 (10.83)	109.35 (12.87)	101.91 (10.63)	106.89 (12.24)	109.21 (11.61)	113.08 (12.93)
Diastolic BP (mmHg) *	65.82 (8.73)	70.02 (9.61)	65.58 (8.69)	68.82 (9.48)	69.65 (8.59)	71.85 (9.54)
Glucose (mg/dl) *	91.56 (9.46)	99.61 (19.78)	91.41 (9.41)	97.23 (15.47)	93.80 (9.90)	103.22 (24.53)
Total cholesterol (mg/dl) *	191.39 (29.65)	200.91 (31.53)	190.93 (29.66)	200.64 (30.17)	198.58 (28.46)	201.32 (33.54)

LDL-C (mg/dl) *	116.30 (27.13)	132.36 (30.07)	115.61 (27.03)	131.32 (28,71)	127.03 (26.46)	133.92 (32.00)
HDL-C (mg/dl) *	68.81 (15.17)	56.13 (13.54)	69.21 (15.15)	57.54 (13.89)	62.49 (14.24)	53.99 (12.71)
Triglycerides (mg/dl) §	77.80(34.52)	119.54 (61.15)	76.90 (33.85)	114.77 (63.57)	92.08 (41.35)	126.80 (56.60)

Data are presented as * mean (± standard deviation), § median (interquartile range), or percentage.
Abbreviations: BMI, body mass index; BP, blood pressure; HDL-C, high-density lipoprotein cholesterol; HEPA, health-enhancing physical activity; LDL-C, low-density lipoprotein cholesterol; NAFLD, nonalcoholic fatty liver disease
†≥10 g ethanol per day
‡ ≥3 times/week
‖ ≥college graduate

Table S2. Cross-sectional association between NAFLD and vasomotor symptoms in lean and overweight premenopausal women after adjusting for confounders including **depression** (n = 4,242)

Vasomotor symptoms			Lean		Overweight	
			NAFLD (−)	NAFLD (+)	NAFLD (−)	NAFLD (+)
Overall	No. of cases		479	45	215	194
	Prevalence (%)		18.89	26.79	23.78	30.60
	Age-adjusted PR* (95% CI)		reference	1.39 (1.08–1.81)	1.24 (1.07–1.42)	1.57 (1.36–1.82)
	Multivariable-adjusted PR*,† (95% CI)	Model 1	reference	1.38 (1.06–1.79)	1.22 (1.06–1.41)	1.49 (1.28–1.73)
		Model 2	reference	1.38 (1.06–1.80)	1.22 (1.06–1.41)	1.49 (1.28–1.73)
Moderate-to-severe	No. of cases		198	24	101	96
	Prevalence (%)		7.81	14.29	11.18	15.14
	Age-adjusted PR* (95% CI)		reference	1.79 (1.21–2.65)	1.40 (1.12–1.76)	1.87 (1.49–2.36)
	Multivariable-adjusted PR*,† (95% CI)	Model 1	reference	1.73 (1.16–2.57)	1.38 (1.10–1.74)	1.74 (1.37–2.21)
		Model 2	reference	1.74 (1.17–2.59)	1.38 (1.10–1.74)	1.75 (1.38–2.22)

*Logistic regression models with robust variance were used to estimate PR and 95% CI for prevalent vasomotor symptoms.

†Multivariable model 1 was adjusted for age; education level; parity; physical activity level; smoking status; alcohol intake; age at menarche; and history of hypertension, diabetes, and dyslipidemia. Multivariable model 2 was adjusted for all the same variables as model 1 plus **depression.**

Abbreviations: CI, confidence interval; NAFLD, nonalcoholic fatty liver disease; PR, prevalence ratio

Table S3. Cross-sectional association between fatty liver and vasomotor symptoms in premenopausal women according to abdominal obesity (n = 4,240)

Vasomotor symptoms		Abdominal non-obese		Abdominal obese	
		NAFLD (–)	NAFLD (+)	NAFLD (–)	NAFLD (+)
Overall	No. of cases	640	131	54	108
	Prevalence (%)	19.79	27.07	26.47	33.96
	Age-adjusted PR* (95% CI)	reference	1.33 (1.13–1.57)	1.31 (1.03–1.66)	1.68 (1.42–1.99)
	Multivariable-adjusted PR*,† (95% CI)	reference	1.23 (1.03–1.47)	1.16 (0.90–1.51)	1.41 (1.15–1.73)
Moderate-to-severe	No. of cases	265	72	34	48
	Prevalence (%)	8.20	14.88	16.67	15.09
	Age-adjusted PR* (95% CI)	reference	1.76 (1.38–2.24)	1.98 (1.43–2.76)	1.80 (1.35–2.39)
	Multivariable-adjusted PR*,† (95% CI)	reference	1.59 (1.21–2.08)	1.73 (1.19–2.51)	1.45 (1.03–2.04)

*Logistic regression models with robust variance were used to estimate PRs and 95% CIs for prevalent vasomotor symptoms.
†The multivariable model was adjusted for age; body mass index; education level; parity; physical activity level; smoking status; alcohol intake; age at menarche; and history of hypertension, diabetes, and dyslipidemia.
Abbreviations: CI, confidence interval; NAFLD, nonalcoholic fatty liver disease; PR, prevalence ratio.

Table S4. Cross-sectional association between fatty liver and vasomotor symptoms (hot flashes or night sweats) in premenopausal women according to abdominal obesity (n = 4,240)

		Abdominal non-obese		Abdominal obese	
		NAFLD (–)	NAFLD (+)	NAFLD (–)	NAFLD (+)
Hot flash	No. of cases	533	113	46	95
	Prevalence (%)	16.48	23.40	22.55	29.87
	Age-adjusted PR* (95% CI)	reference	1.39 (1.16–1.66)	1.35 (1.03–1.76)	1.78 (1.48–2.15)
	Multivariable-adjusted PR* (95% CI)	reference	1.26 (1.03–1.53)	1.15 (0.86–1.50)	1.44 (1.15–1.81)
Night sweat	No. of cases	389	86	35	69
	Prevalence (%)	11.98	17.84	17.24	21.70
	Age-adjusted PR* (95% CI)	reference	1.46 (1.17–1.80)	1.41 (1.03–1.94)	1.78 (1.41–1.23)
	Multivariable-adjusted PR*,† (95%)	reference	1.41 (1.11–1.78)	1.36 (0.95–1.94)	1.59 (1.20–2.01)

*Logistic regression models with robust variance were used to estimate PRs and 95% CIs for prevalent vasomotor symptoms.
*,†The multivariable model was adjusted for age; body mass index; education level; parity; physical activity level; smoking status; alcohol intake; age at menarche; and history of hypertension, diabetes, and dyslipidemia.
Abbreviations: CI, confidence interval; NAFLD, nonalcoholic fatty liver disease; PR, prevalence ratio.

Table S5. Cross-sectional association between nonalcoholic fatty liver disease and vasomotor symptoms in premenopausal women according to body fat percentage (n = 4,231)

Vasomotor symptoms		Body fat percentage <35%		Body fat percentage ≥35%	
		NAFLD (−)	NAFLD (+)	NAFLD (−)	NAFLD (+)
Overall	No. of cases	574	102	120	137
	Prevalence (%)	19.31	26.63	25.70	32.70
	Age-adjusted PR* (95% CI)	Reference	1.33 (1.11–1.60)	1.32 (1.11–1.57)	1.67 (1.43–1.95)
	Multivariable-adjusted PR*,† (95% CI)	Reference	1.24 (1.01–1.51)	1.22 (1.00–1.49)	1.47 (1.21–1.79)
Moderate-to-severe	No. of cases	237	55	62	65
	Prevalence (%)	7.98	14.36	13.28	15.51
	Age-adjusted PR* (95% CI)	Reference	1.73 (1.31–2.27)	1.65 (1.27–2.14)	1.91 (1.48–2.46)
	Multivariable-adjusted PR*,† (95% CI)	Reference	1.56 (1.15–2.12)	1.46 (1.08–1.99)	1.60 (1.16–2.21)

*Logistic regression models with robust variance were used to estimate PR and 95% CI for prevalent vasomotor symptoms.

†The multivariable model was adjusted for age; body mass index; education level; parity; physical activity level; smoking status; alcohol intake; age at menarche; and history of hypertension, diabetes, and dyslipidemia

Abbreviations: CI, confidence interval; NAFLD, nonalcoholic fatty liver disease; PR, prevalence ratio

Table S6. Cross-sectional association between nonalcoholic fatty liver disease with or without intermediate-to-high probability of advanced fibrosis based on the Fibrosis-4 index and vasomotor symptoms in premenopausal women according to general obesity (n = 4,232)

		Lean			Overweight		
		NAFLD (−) (n=2,536)	NAFLD plus low FIB-4 (n=162)	NAFLD plus Intermediate/high FIB-4 (n=6)	NAFLD (−) (n=904)	NAFLD plus low FIB-4 (n=617)	NAFLD plus Intermediate/high FIB-4 (n=17)
Overall VMS	No. of cases	479	43	2	215	186	8
	Prevalence (%)	18.89	26.54	33.33	23.78	30.15	47.06
	Age-adjusted PR* (95% CI)	Reference	1.38 (1.06–1.81)	1.58 (0.51–4.87)	1.24 (1.07–1.42)	1.55 (1.34–1.79)	2.39 (1.45–3.95)
	Multivariable-adjusted PR*,† (95% CI)	Reference	1.37 (1.05–1.79)	1.46 (0.47–4.50)	1.22 (1.06–1.41)	1.46 (1.26–1.70)	2.33 (1.40–3.89)

*Logistic regression models with robust variance were used to estimate PR and 95% CI for prevalent vasomotor symptoms.

†The multivariable model was adjusted for age, education level, parity, physical activity level, smoking status, alcohol intake, age at menarche, and history of hypertension and diabetes.

Abbreviations: CI, confidence interval; FIB-4, Fibrosis-4 index; NAFLD, nonalcoholic fatty liver disease; PR, prevalence ratio; VMS, vasomotor symptoms

Table S7. Longitudinal association between fatty liver and vasomotor symptoms in premenopausal women according to adiposity measures

Type	Person-years (PY)	No. of cases	Incident rate (cases per 100 PY)	Age-adjusted HR (95% CI)	Multivariable-adjusted HR (95% CI)
**General obesity** (n = 2,349)					
Lean - NAFLD (–)	6149.65	579	9.42	reference	reference
Lean - NAFLD (+)	358.33	39	10.88	1.15 (0.83-1.59)	1.19 (0.85-1.65)
Overweight - NAFLD (–)	1956.87	232	11.86	1.28 (1.09-1.49)	1.28 (1.09-1.49)
Overweight - NAFLD (+)	1194.82	152	12.72	1.30 (1.09-1.56)	1.31 (1.09-1.59)
**Abdominal obesity** (n = 2,348)					
Nonobese - NAFLD (–)	7645.51	748	9.78	reference	reference
Nonobese - NAFLD (+)	964.2	123	12.76	1.30 (1.07-1.58)	1.19 (0.96-1.47)
Obese - NAFLD (–)	455.1	62	13.62	1.42 (1.09-1.85)	1.26 (0.94-1.69)
Obese - NAFLD (+)	588.95	68	11.55	1.09 (0.85-1.41)	0.98 (0.74-1.31)
**Body fat percentage** (n = 2,349)					
<35% - NAFLD (–)	7140.59	703	9.85	reference	reference
<35% - NAFLD (+)	788.09	98	12.44	1.22 (0.99-1.52)	1.11 (0.88-1.40)
≥35% - NAFLD (–)	965.93	108	11.18	1.17 (0.95-1.44)	1.06 (0.84-1.34)

≥35% - NAFLD (+)	765.06	93	12.16	1.20 (0.96-1.49)	1.07 (0.83-1.38)

Abbreviations: CI, confidence interval; HR, hazard ratio; VMS, vasomotor symptoms.

*Parametric proportional hazard models were used. The multivariable model was adjusted for age; education level; parity; physical activity level; smoking status; alcohol intake; age at menarche; and history of hypertension, diabetes, dyslipidemia, and body mass index (abdominal obesity and body fat percent only)

Figure S1. Flow chart of the study population (n = 2,349)

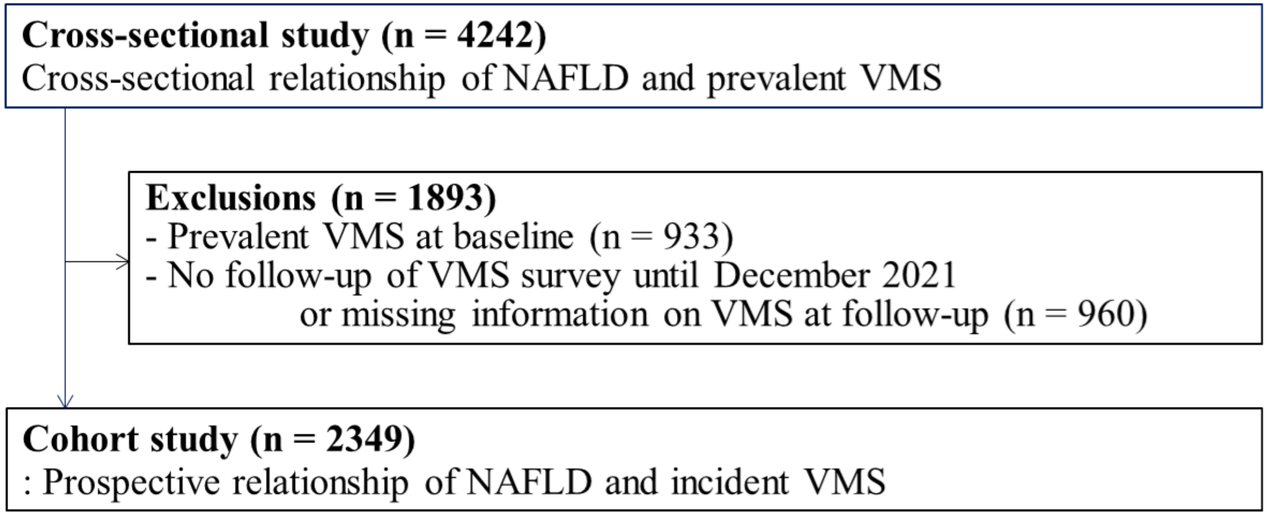

Supplement: Supplementary file 1 [file nutrients-14-02805-s001.zip › nutrients-1791526-supplementary.pdf]
